# A Rare Case of Cementoblastoma of the Second Right Maxillary Premolar in a 30-Year-Old Man

**DOI:** 10.7759/cureus.73737

**Published:** 2024-11-15

**Authors:** Simeon N Dimanov, Alexandar L Stoev, Ralitsa V Yotsova, Lyuben L Stoev, Yanko G Yankov, Yoana R Ruseva

**Affiliations:** 1 Department of Oral Surgery, Medical University "Prof. Dr. Paraskev Stoyanov", Varna, BGR; 2 Faculty of Dentistry, Medical University "Prof. Dr. Paraskev Stoyanov", Varna, BGR; 3 Department of General and Clinical Pathology, Forensic Medicine and Deontology, Medical University "Prof. Dr. Paraskev Stoyanov", Varna, BGR; 4 Clinic of Maxillofacial Surgery, University Hospital "St. Marina", Varna, BGR; 5 Department of General and Operative Surgery, Medical University "Prof. Dr. Paraskev Stoyanov", Varna, BGR; 6 Department of Pediatric Dentistry, Medical University "Prof. Dr. Paraskev Stoyanov", Varna, BGR

**Keywords:** benign tumor, cementoblastoma, jaw tumor, maxilla, maxillofacial surgery, odontogenic neoplasm, odontogenic tumor, oral surgery, partial maxillectomy, upper jaw

## Abstract

Cementoblastoma is a benign odontogenic mesenchymal tumor characterized by cementum production. Cementoblastoma is considered a relatively rare neoplasm with a predilection to the posterior region of the mandible. The main clinical differential diagnoses include hypercementosis, cemento-osseous dysplasia, condensing osteitis, idiopathic osteosclerosis, osteoblastoma, odontoma, and osteosarcoma. Imaging findings may be pathognomonic when demonstrative. Although rather identical histologically, cementoblastoma exhibits unequivocal fusion to the root of the tooth, which distinguishes it from osteoblastoma. We present a case of a 30-year-old man with cementoblastoma arising in an unusual location: the root of the second right premolar of the maxilla.

## Introduction

Cementoblastoma is a benign odontogenic tumor of mesenchymal origin presenting with a cementum-like mass that is attached to the root of a tooth. Cementoblastoma accounts for up to 3% of all odontogenic neoplasms and is more prevalent in young people. It most commonly affects the posterior region of the mandible [1,2].

Cementoblastoma demonstrates high growth potential and a recurrence rate of up to 37%. Its recurrence is usually associated with incomplete tumor removal. The clinical findings vary from small asymptomatic lesions to painful, rapidly growing masses, causing jaw expansion and destruction, tooth mobility, malocclusion, erosions, bleeding, pathological fractures, and nerve damage. The most common radiological appearance is a radiopaque formation with a radiolucent rim. Diagnosis is based on the clinical and radiological features and the histopathological report. Cone-beam computed tomography (CBCT) is an important diagnostic method used for evaluating jaw pathology and displaying three-dimensional orientation, size, and tooth involvement. The differential diagnoses include most odontogenic and nonodontogenic jaw tumors. The histopathological appearances of cementoblastoma and osteoblastoma are almost indistinguishable, so the connection between the lesion and the periodontal ligament is essential for the correct diagnosis [3,4].

Conservative approaches such as apical resection and curettage have been associated with recurrence. The optimal treatment protocol involves resection with a border of healthy tissues and the removal of the corresponding tooth [4].

This case report presents a relatively rare case of cementoblastoma of the second right premolar of the upper jaw and our experience in treating this disease.

## Case presentation

**Figure 1:**
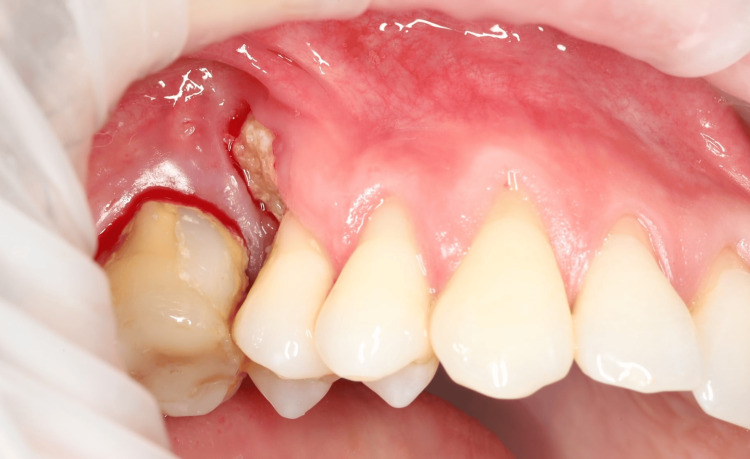
Intraoral view of the lesion of the upper jaw attached to tooth 15

This case report describes a 30-year-old man who was admitted to a private specialized dental clinic for oral surgery in the city of Varna, Bulgaria, at the end of June 2024. He had been referred to the clinic by a doctor of dental medicine because he had had a hard, asymptomatic cementum-like lesion on the maxilla attached to the root of tooth 15 for approximately 12 years (Figure 1).

The patient did not have any complaints. The patient had no general diseases, no allergies, and no regular intake of any medications. He had undergone a surgical procedure, amputation, 15 years ago for a cyst-like formation on the mesial root of tooth 16, for which the patient does not provide any more information.

The clinical examination revealed a diffuse cementum-like hard lesion at the buccal side of the alveolar crest above tooth 15. The lesion penetrated the mucosa and adhered to the root of tooth 15. The overlying mucosa showed no signs of infection, but the gingiva of tooth 15 showed signs of recession.

**Figure 2:**
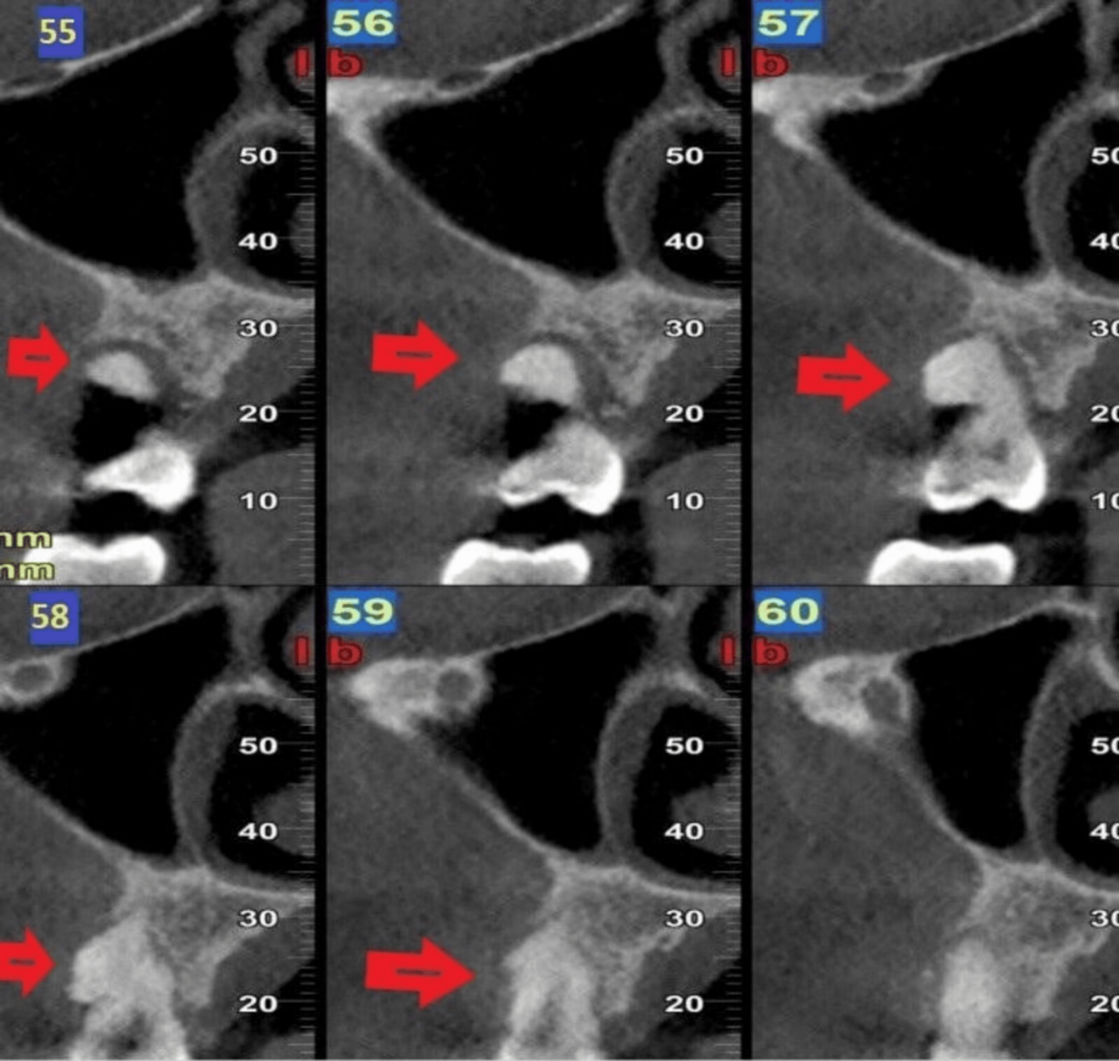
Images 55, 56, 57, 58, and 59 of CBCT paraxial cuts from distal to medial showing the lesion Images 55, 56, 57, 58, and 59 of CBCT paraxial cuts from distal to medial showing the lesion diameter and attachment to the second premolar on the upper right jaw (red arrows). The capsule of the tumor is also visible. The tumor was not visible on image 60. Here, “l” indicates lingual, and “b” represents buccal, which help the clinician to determine anatomical and pathoanatomical structures CBCT: cone-beam computed tomography

An initial panoramic radiograph demonstrated a round radiopaque mass with mixed radiodensity surrounded by a radiolucent rim, which is easy to discern within the nonmineralized radiolucencies that surround the tumor in the growth zone. The lesion was attached to the root of the second right premolar of the upper jaw. Cone-beam computed tomography (CBCT) images demonstrated a well-circumscribed mass involving the root of the maxillary second right premolar (Figure 2). Our differential diagnosis included a benign tumor of the maxilla, calcifying epithelial odontogenic tumor, and ossifying fibroma.

**Figure 3:**
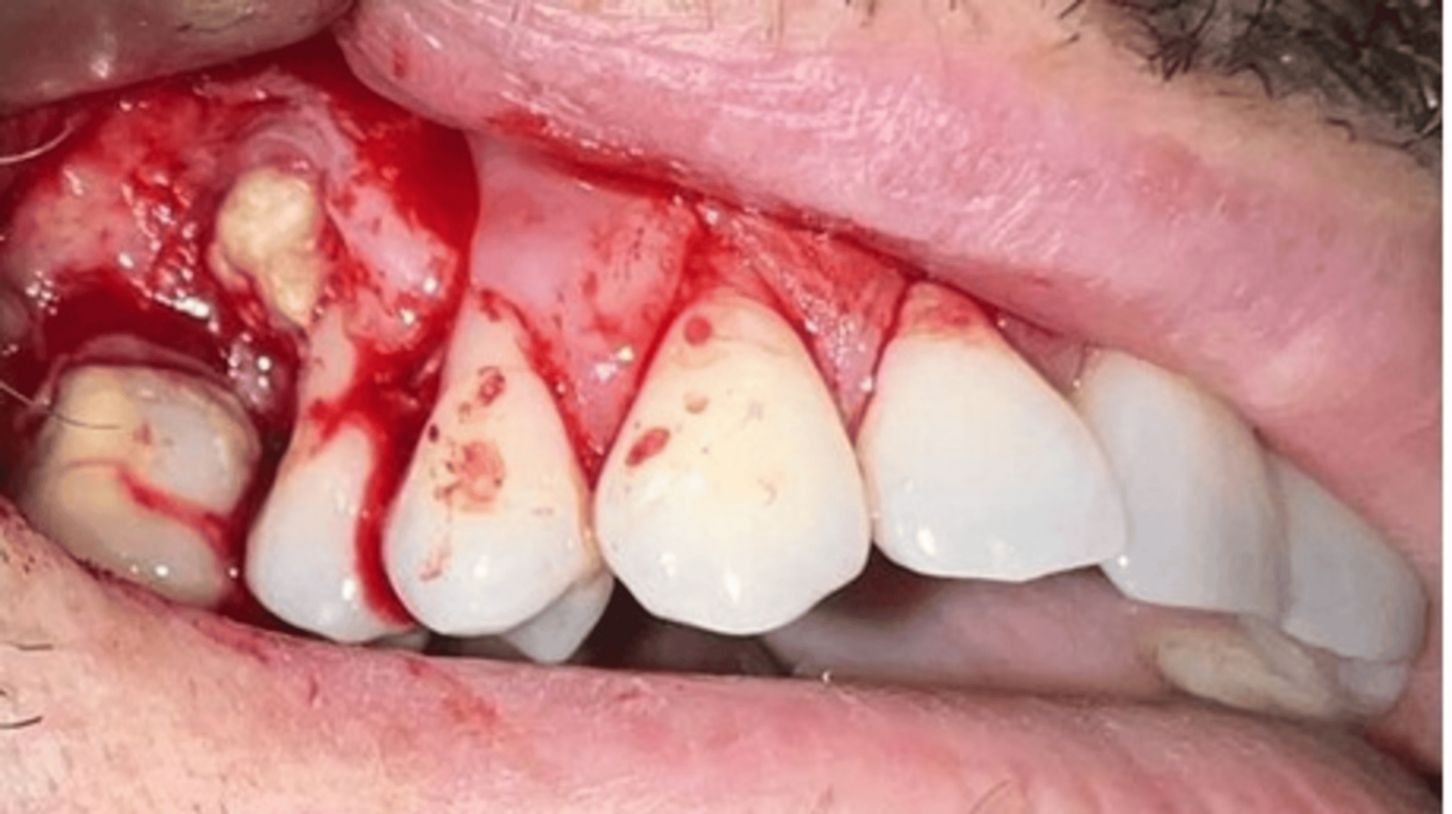
Intraoperative view of the tumor attached to tooth 15 after the mucoperiosteal flap was raised

**Figure 4:**
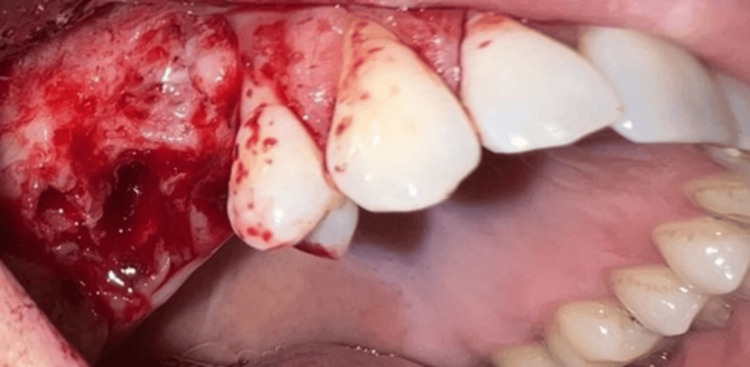
Intraoperative view of the bony defect after excision of the tumor and extracting the maxillary first molar and second premolar on the upper right jaw

**Figure 5:**
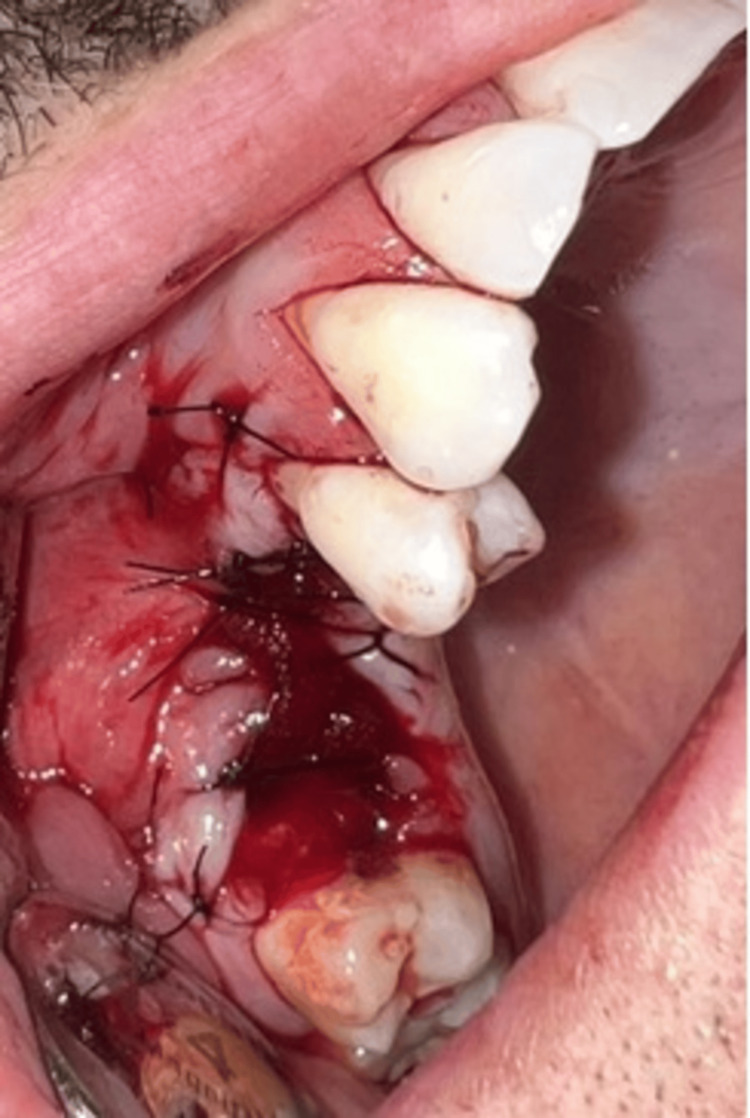
The wound was closed, and sutures were placed

Our treatment plan included the following: bone resection with a border of 5 mm, with extraction of the second upper right premolar, extraction of the first upper right molar, histopathological analysis, and follow-up of the patient. The surgical intervention was performed under meticulous antiseptic of the operative field with Braunol 7.5 g/100 g, solution Povidone, iodinated, and local infiltrative anesthesia with 4% articaine, 2.5 ml. A trapezoidal mucoperiosteal flap on the upper nutrient base was raised on the vestibular side of the maxilla in the region of teeth 17-14 (Figure 3). The tumor was excised en bloc with the extraction of tooth 15 with a clearance margin of 5 mm. The tumor, with a diameter of 13 mm, was excised. Both the extracted tooth 15 and the tumor were placed in a 10% formalin solution and sent for further histological examination. Tooth 16 was separately extracted because of periodontitis chronica granulomatosa localisata. Bone resection around the borders of the tumor was performed with a 5 mm surgical border (Figure 4). Collagen cones were placed inside the osteotomy defect. Conservative approaches, such as enucleation, allow for guided bone regeneration (GBR) at the surgical site [5]. However, as the risk of recurrence increases with non-radical methods, GBR was not performed in this case. Hemostasis was applied. The flap was mobilized, adapted, and sutured with a 4/0 non-resorbable polyfilament (Figure 5).

The postoperative medication therapy included antibiotics (amoxicillin + clavulanic acid three times daily per 1 gram peroral intake) and analgesics (nimesulide two times daily per 0.1 gram peroral intake). The wound was locally treated by the patient with a 0.12% chlorhexidine mouth rinse (EluPerio) twice per day for two weeks.

Checkups were conducted on the seventh, tenth, and fourteenth postoperative days.

**Figure 6:**
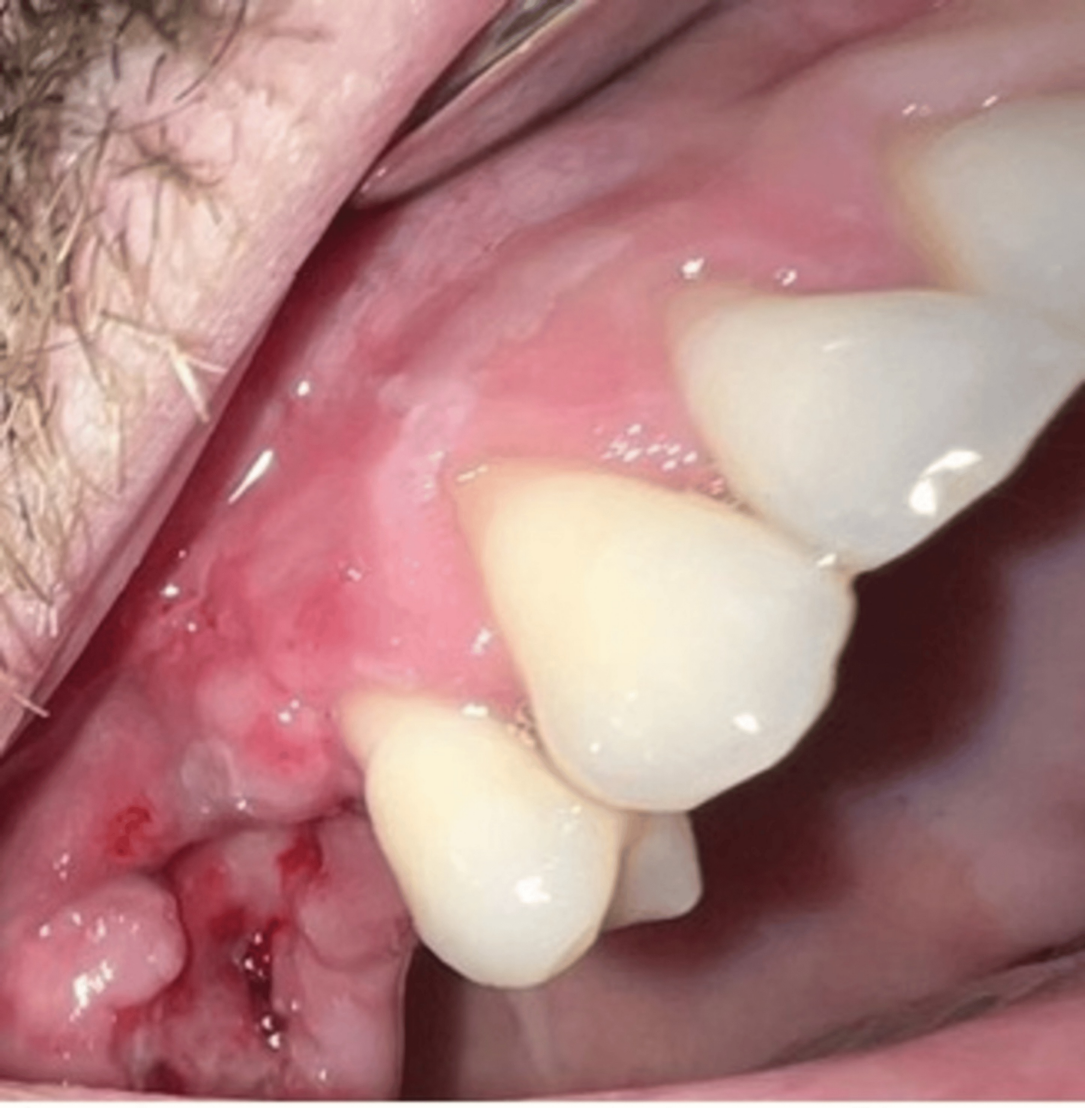
Intraoral view of the wound after the removal of the sutures on the 10th postoperative day, showing a normal healing process

On the seventh day during the checkup, the patient had no complaints; the healing was uneventful, without suppuration or bleeding. The sutures were relaxed without tension on the 10th postoperative day (Figure 6).

The postoperative period was smooth, without notable edema, pain, or other complaints. Sutures were removed on the tenth postoperative day. During the checkup, the wound was without any bleeding, suppuration, or signs of infection; no symptoms were detected; and no complaints from the patient were declared.

The last postoperative checkup was conducted on the fourteenth day. The patient again had no complaints, and the wound was epithelized and normotrophic.

**Figure 7:**
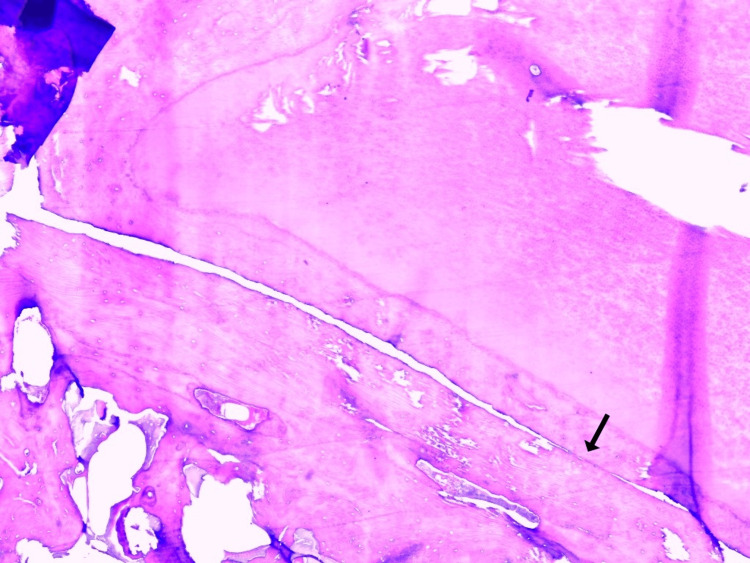
The lesion was continuous with the root of the tooth (arrow) (H&E, 4x) H&E: Hematoxylin and Eosin

**Figure 8:**
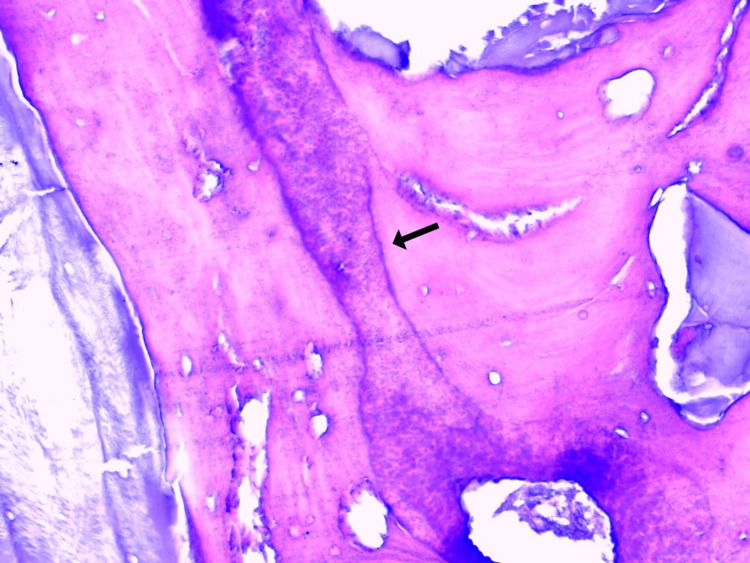
Photomicrograph showing numerous prominent basophilic reversal lines (arrow) (H&E, 20x) H&E: Hematoxylin and Eosin

The histopathological examination revealed a hypocellular lesion composed of cementum with trabecular arrangement and prominent basophilic reversal lines. The mass showed clear attachment to the root of the tooth (Figures 7, 8). The morphology was compatible with cementoblastoma.

**Figure 9:**
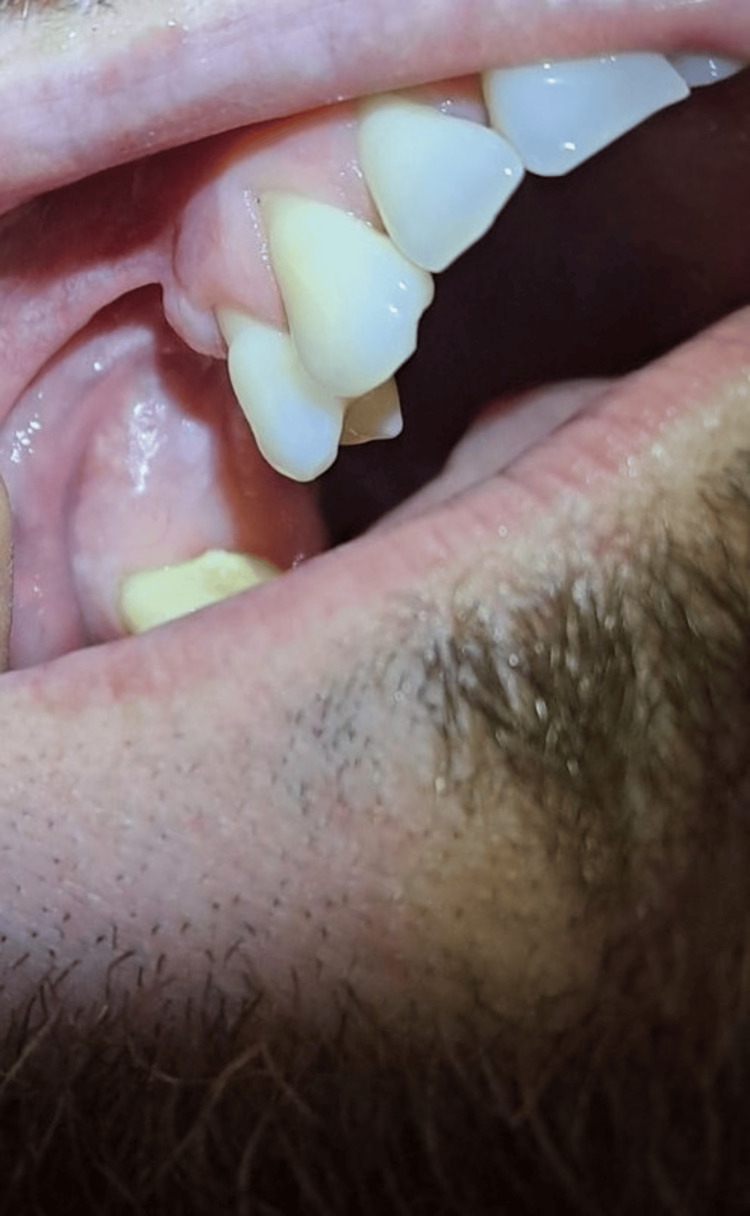
Clinical photograph at the three-month follow-up, showing proper healing of the surgical site

A control checkup was conducted three months after the surgery. We found no clinical recurrence, and the mucosa in the region was anatomically normal (Figure 9).

## Discussion

The first complete description of cementoblastoma was reported in 1889 by Metnitz, a professor of dentistry at the University of Vienna, Austria, although under the term “odontoma” [6]. Three decades later, Dewey described cementoblastoma as an odontogenic tumor with a mesenchymal origin [7]. Bland-Sutton published the first radiographic presentation of cementoblastoma in 1906 [6].

According to the 2022 World Health Organization (WHO) classification, cementoblastoma is a benign mesenchymal odontogenic tumor, along with odontogenic fibroma, cemento-ossifying fibroma, and odontogenic myxoma [8,9].

Cementoblastoma most commonly affects young patients during their second or third decade of life, with 20 years being the mean age of cementoblastoma presentation, although some authors have reported cases associated with deciduous teeth [7,10]. Our case is similar: the tumor formation was present for 12 years when the patient was in his early twenties. Cementoblastoma is characterized by no sex predilection, but a higher prevalence has been reported in women. However, the differences were interpreted as statistically not significant [8,10]. Other authors reported cementoblastoma as being more common in men, with a male/female ratio of 1.2:1 [11].

Cementoblastoma occurs more commonly in the mandible (93%) than in the maxilla (7%), mainly in the mandibular posterior region, affecting the apical third of the permanent first molar as well as the second premolar [8,10], unlike our presented case, where the tumor mass was associated with the maxillary second premolar on the right side. The tumor lesion is more commonly found on the right side of the mandibular arch (71.5%), followed by its left side (21.5%), and the right side of the maxillary molar region (7%). An incisor location of cementoblastoma is extremely rare [10].

Three major stages are involved in the pathogenesis of cementoblastoma. The process starts with periapical osteolysis and is followed by a cementoblastic stage. The third stage is characterized by calcification and maturation [12]. This cementum-like calcified tissue fuses with the vital permanent tooth root and presents as a localized hard mass with cortical expansion, as was the case in the patient we describe: the vital permanent tooth affected was the second premolar on the right side. Other conditions affecting the teeth and the surrounding tissues (chronic focal sclerosing osteitis, hypercementosis, and osteoblastoma) also present with osteocementum-like material production and should be considered in the differential diagnosis because of the similarities in the clinical features. A final diagnosis is impossible without a histopathological evaluation [13].

Lam et al. reported that cementoblastomas and osteoblastomas share a common molecular pathogenesis that is based on the occurrence of FOS gene rearrangements and FOS overexpression, suggesting that both entities form a spectrum of the same disease localized at the tooth root. The FOS gene is part of the FOS gene family, which is involved in the formation of transcription factor complex activator protein 1. FOS protein binds to the promoter and enhancer regions of target genes and regulates cell proliferation, differentiation, and transformation. The proteins from this family are highly expressed during normal osteoblast maturation. FOS gene rearrangements are found in epithelioid hemangioma and osteoid-osteoma/osteoblastoma (with c-FOS overexpression), demonstrating the important role of this gene in not only normal physiological processes but also pathological conditions [14].

Clinically, cementoblastoma may be asymptomatic, or usually in more than 70% of the cases, according to Chrcanovic et al., is present as a painful swelling in the affected area of the mandibular or maxillary alveolar bone [11,12]. Other common findings include bone expansion and facial asymmetry. Rarely reported symptoms and findings are lip-paresthesia, pathological jaw fracture, as well as cortical bone perforation, and inferior displacement of the mandibular canal. In addition to tooth resorption, the vitality of the tooth usually remains preserved [10]. Our patient did not have any of the listed symptoms above and had the lesion for twelve years without any symptoms.

Cementoblastoma presents upon radiological examination as a radiopaque mass confluent with the root of the affected tooth and surrounded by a thin radiolucent rim [11,15]. In addition, root resorption and obliteration of the periodontal ligament space are commonly found, but not in our patient’s case; he only experienced radiopaque formation connected to the maxillary second right premolar with the characteristic radiolucent rim [16].

Histopathologically, cementoblastoma is composed of cell-poor cementum with a fibrovascular stromal background [17]. Classically, the cementum shows substantial cementoblastic rimming; the presence of multinucleated osteoclast-like giant cells in the surrounding stroma is common. The vast majority of the lesions show accentuated reversal lines, which impart the cementoblastoma a Pagetoid appearance. Mitotic activity is lacking despite the possible pleomorphism of the lining cementoblasts and giant cells; this is a major feature in the differential diagnosis of osteosarcoma [18]. The histological appearances of osteoblastoma and cementoblastoma are identical, with tooth root attachment being the only distinguishing trait [17,18]. The histopathological findings described in the present case are consistent with most of the classical features, especially with the much-accentuated reversal lines; prominent osteoblastic rimming is not evident because of the decalcifying artifact.

The essential diagnostic criteria from imaging studies for cementoblastoma include the presence of a mass fused to a tooth root that is densely mineralized with a radiating peripheral matrix, plump cementoblasts, and no fibro-osseous component found upon histological examination, which does not align with the case we presented [8]. The direct connection of cementoblastoma with the tooth's radicular surface is the most important imaging finding, according to the WHO [12].

The suggested treatment for cementoblastoma involves excising the lesion along with the corresponding tooth or teeth and then performing curettage or peripheral ostectomy. The recurrence rate of this neoplasm is the highest for those treated with curettage alone. Benign cementoblastomas can uncontrollably grow, which is why some specialists prefer extraction followed by curettage to eliminate the chance of recurrence [18]. The recurrence rate of cementoblastoma broadly varies between 11.8% and 37.1% [10]. Usually diagnosed within six months to one year after surgery, recurrence episodes are more common when curettage is performed without the extraction of the involved tooth or teeth [12]. We chose bone resection with a border of 5 mm with extraction of the second upper right premolar to avoid recurrence. Some authors recommended root apicectomy for cases when the tooth is preserved because the cementoblasts in the apical third of the root might produce a matrix at an uncontrolled rate, favoring cementoblastoma recurrence [19].

## Conclusions

Cementoblastoma is a benign cement-like odontogenic tumor affecting the jaw. It is more common in young patients and at the molar site of the lower jaw. The reported case presented with an asymptomatic cementoblastoma in a relatively rare location: the premolar area of the upper jaw. The appropriate diagnostic methods, including CBCT imaging and histopathological evaluation, are essential for clinical decision-making. Resection with a border of healthy tissue is the treatment of choice and reduces the risk of recurrence. This case report highlights the possible variability in the predilection sites for cementoblastoma, which can cause some diagnostic and treatment mistakes and, thus, increase the risks of recurrence. Understanding this variability can reduce misdiagnosis and guide clinicians to use the correct treatment modalities.
